# Hygienic Resort

**Published:** 1897-01

**Authors:** 


### Hygienic Resort.

A most excellent place for recuperation, for building up a worn and exhausted physical system, is to be found at "Hot Springs, Va." The medical qualities of these waters are not excelled by any in this country. For nervous diseases they are eminently efficacious; rheumatism is, in many cases, greatly relieved by their use, and many atime wholly relieved; affections of the digestive system are usually promptly relieved by the use of these waters.

The situation is a beautiful one, being in the mountain regions, two thousand feet above sea level, and the air clear and pure.

Most complete accommodations are provided for visitors and temporary residents. The place is very accessible, easily reached from all parts of the country. A residence there of from one to three weeks will be invaluable to the exhausted dentist.

